# Supplementary material for: Statistical Machine-Learning Methods for Genomic Prediction Using the SKM Library
Source: Genes (Basel). 2023 Apr 28;14(5):1003. doi: 10.3390/genes14051003 (PMC10218433; doi:10.3390/genes14051003)
Supplement: Supplementary file 1 [file genes-14-01003-s001.zip › genes-2343983-supplementary.pdf]

Supplementary Material

Example S1. Univariate continuous variable

```
# Import SKM library
library(SKM)
# Load the dataset
load("Japonica_100.RData", verbose = TRUE)

# Oder Pheno by Env and Lines
Pheno <- Pheno[order(Pheno$Env, Pheno$Line), ]
Markers=Markers
geno_order <- sort(rownames(Markers))
Markers=Markers[geno_order ,]
Markers_Scale=scale(Markers[,-1])
dim(Markers_Scale)
Markers_Scale=Markers_Scale[ , colSums(is.na(Markers_Scale))==0]
dim(Markers_Scale)
Geno=Markers_Scale%*%t(Markers_Scale)/ncol(Markers_Scale)

# Order Geno by Line
Geno <- Geno[geno_order, geno_order]
Markers_SVD=svd(Markers_Scale)
dim(Markers_SVD$u)
Markers_Red=Markers_SVD$u%*%diag(Markers_SVD$d)
dim(Markers_Red)
# Design matrices
Z_g<- model.matrix(~ 0 + Line, data = Pheno)
K_g=Z_g%*% Geno%*%t(Z_g) ###Linear kernel

# Env design matrix without the first column
X_E<- model.matrix(~ 0 + Env, data = Pheno)[, -1]
K_e=X_E%*%t(X_E)/ncol(X_E)

X_g <-Z_g%*%Markers_Red
K_ge <- K_e* K_g
# Interaction matrix
X_ge<- model.matrix(~ 0 + X_g:X_E, data = Pheno)
dim(X_ge1)
# Bind all matrices in a single one
X <- cbind(X_E, X_g, X_ge)

# Retrieve the continuos response variable
y <- as.numeric(Pheno$GY)

# Folds generation for random line validation
folds <- cv_random_line(lines =as.character(Pheno$Line),folds_number=5,
testing_proportion =0.2)
#

# Data frame for storing the individual predictions at each fold
```

```
Predictions <- data.frame()

for (i in seq_along(folds)) {
  fold <- folds[[i]]

  # Split training and testing data
  X_training <- X[fold$training, ]
  X_testing <- X[fold$testing, ]
  y_training <- y[fold$training]
  y_testing <- y[fold$testing]

  # *********** MODEL EVALUATION AND HYPERPARAMETERS TUNING WITH SKM
***********
  model <- SKM::random_forest(
    X_training,
    y_training,
    trees_number = c(100, 200),
    node_size = c(5, 2),

    tune_type = "Grid_search",
    tune_cv_type = "k_fold",
    tune_folds_number = 5,
    tune_loss_function = "mse"
  )

  # Predict over testing set
  predictions <- predict(model, X_testing)

  # Save the predictions along with environment and line information
  Predictions <- rbind(
    Predictions,
    data.frame(
      Fold = i,
      Line = Pheno$Line[fold$testing],
      Env = Pheno$Env[fold$testing],
      Observed = y_testing,
      Predicted = predictions$predicted
    )
  )
}

# Errors metrics for prediction performance
Summaries <- SKM::gs_summaries(Predictions)

# Print summaries by line, environment and fold
#Summaries$line
Summaries$env
#Summaries$fold
```

Example S2. Univariate binary variable

```
# Import SKM library
library(SKM)

# Load the dataset
load("Japonica_100.RData", verbose = TRUE)

# Oder Pheno by Env and Lines
Pheno <- Pheno[order(Pheno$Env, Pheno$Line), ]
Markers=Markers
geno_order <- sort(rownames(Markers))
Markers=Markers[geno_order ,]
Markers_Scale=scale(Markers[,-1])
dim(Markers_Scale)
Markers_Scale=Markers_Scale[ , colSums(is.na(Markers_Scale))==0]
dim(Markers_Scale)
Geno=Markers_Scale%*%t(Markers_Scale)/ncol(Markers_Scale)

# Order Geno by Line
Geno <- Geno[geno_order, geno_order]
Markers_SVD=svd(Markers_Scale)
dim(Markers_SVD$u)
Markers_Red=Markers_SVD$u%*%diag(Markers_SVD$d)
dim(Markers_Red)
# Design matrices
Z_g<- model.matrix(~ 0 + Line, data = Pheno)
K_g=Z_g%*% Geno%*%t(Z_g) ###Linear kernel

# Env design matrix without the first column
X_E<- model.matrix(~ 0 + Env, data = Pheno)[, -1]
K_e=X_E%*%t(X_E)/ncol(X_E)

X_g <-Z_g%*%Markers_Red
K_ge <- K_e* K_g
# Interaction matrix
X_ge<- model.matrix(~ 0 + X_g:X_E, data = Pheno)
dim(X_ge)
# Bind all matrices in a single one
X <- cbind(X_E, X_g, X_ge)
# Retrieve the binary response variable
y=ifelse(Pheno$GY>quantile(Pheno$GY,probs=0.7),"Top","Bottom")
y <- as.factor(y)
# Folds generation for random line cross validation
folds <- SKM::cv_kfold_strata(
 y,
  k = 5
)

# Data frame for storing the individual predictions at each fold
```

```
Predictions <- data.frame()

for (i in seq_along(folds)) {
  fold <- folds[[i]]

  # Split training and testing data
  X_training <- X[fold$training, ]
  X_testing <- X[fold$testing, ]
  y_training <- y[fold$training]
  y_testing <- y[fold$testing]

  # *********** MODEL EVALUATION AND HYPERPARAMETERS TUNING WITH SKM
***********
  model <- SKM::support_vector_machine(
    X_training,
    y_training,

    kernel = "radial",
    gamma = list(min = 1/ncol(X), max = 0.1),
    cost = list(min = 0.5, max = 1),

    tune_type = "Bayesian_optimization",
    tune_bayes_samples_number = 5,
    tune_bayes_iterations_number = 5,

    tune_cv_type = "random",
    tune_folds_number = 5,
    tune_testing_proportion = 0.25,
    tune_loss_function = "roc_auc"
  )

  # Predict over testing set
  predictions <- predict(model, X_testing)

  # Save the predictions along with environment and line information
  Predictions <- rbind(
    Predictions,
    data.frame(
      Fold = i,
      Line = Pheno$Line[fold$testing],
      Env = Pheno$Env[fold$testing],
      Observed = y_testing,
      Predicted = predictions$predicted,
      predictions$probabilities
    )
  )
}

# Errors metrics for prediction performance
Summaries <- SKM::gs_summaries(Predictions)
```

```
# Print summaries by line, environment and fold
Summaries$line
Summaries$env
Summaries$fold
```

Example S3. Univariate categorical variable

```
# Import SKM library
library(SKM)

# Load the dataset
load("Japonica_100.RData", verbose = TRUE)

# Oder Pheno by Env and Lines
Pheno <- Pheno[order(Pheno$Env, Pheno$Line), ]
Markers=Markers
geno_order <- sort(rownames(Markers))
Markers=Markers[geno_order ,]
Markers_Scale=scale(Markers[,-1])
dim(Markers_Scale)
Markers_Scale=Markers_Scale[ , colSums(is.na(Markers_Scale))==0]
dim(Markers_Scale)
Geno=Markers_Scale%*%t(Markers_Scale)/ncol(Markers_Scale)

# Order Geno by Line
Geno <- Geno[geno_order, geno_order]
Markers_SVD=svd(Markers_Scale)
dim(Markers_SVD$u)
Markers_Red=Markers_SVD$u%*%diag(Markers_SVD$d)
dim(Markers_Red)
# Design matrices
Z_g<- model.matrix(~ 0 + Line, data = Pheno)
K_g=Z_g%*% Geno%*%t(Z_g) ###Linear kernel

# Env design matrix without the first column
X_E<- model.matrix(~ 0 + Env, data = Pheno)[, -1]
K_e=X_E%*%t(X_E)/ncol(X_E)

X_g <-Z_g%*%Markers_Red
K_ge <- K_e* K_g
# Interaction matrix
X_ge<- model.matrix(~ 0 + X_g:X_E, data = Pheno)
dim(X_ge)
# Bind all matrices in a single one
X <- cbind(X_E, X_g, X_ge)

# Retrieve the categorical response variable
```

```
y=ifelse(Pheno$GY<quantile(Pheno$GY,probs=0.3),"Bottom",ifelse(Pheno$GY>q
uantile(Pheno$GY,probs=0.7),"Top","Middle"))
y <- as.factor(y)

# Folds generation for leave one environmet out cross validation
folds <- SKM::cv_leve_one_group_out(as.character(Pheno$Env))

# Data frame for storing the individual predictions at each fold
Predictions <- data.frame()

for (i in seq_along(folds)) {
  fold <- folds[[i]]

  # Split training and testing data
  X_training <- X[fold$training, ]
  X_testing <- X[fold$testing, ]
  y_training <- y[fold$training]
  y_testing <- y[fold$testing]

  # *********** MODEL EVALUATION AND HYPERPARAMETERS TUNING WITH SKM
***********
  model <- SKM::generalized_linear_model(
    X_training,
    y_training,

    alpha = c(0),

    tune_type = "Grid_search"
  )

  # Predict over testing set
  predictions <- predict(model, X_testing)

  # Save the predictions along with environment and line information
  Predictions <- rbind(
    Predictions,
    data.frame(
      Fold = i,
      Line = Pheno$Line[fold$testing],
      Env = Pheno$Env[fold$testing],
      Observed = y_testing,
      Predicted = predictions$predicted,
      predictions$probabilities
    )
  )
}

# Errors metrics for prediction performance
Summaries <- SKM::gs_summaries(Predictions)
```

```
# Print summaries by line, environment and fold
Summaries$line
Summaries$env
Summaries$fold
```

Example S4. Multivariate continuous variables

```
# Import SKM library
library(SKM)

# Load the dataset
load("Japonica_100.RData", verbose = TRUE)

# Oder Pheno by Env and Lines
Pheno <- Pheno[order(Pheno$Env, Pheno$Line), ]
Markers=Markers
geno_order <- sort(rownames(Markers))
Markers=Markers[geno_order ,]
Markers_Scale=scale(Markers[,-1])
dim(Markers_Scale)
Markers_Scale=Markers_Scale[ , colSums(is.na(Markers_Scale))==0]
dim(Markers_Scale)
Geno=Markers_Scale%*%t(Markers_Scale)/ncol(Markers_Scale)

# Order Geno by Line
Geno <- Geno[geno_order, geno_order]
Markers_SVD=svd(Markers_Scale)
dim(Markers_SVD$u)
Markers_Red=Markers_SVD$u%*%diag(Markers_SVD$d)
dim(Markers_Red)
# Design matrices
Z_g<- model.matrix(~ 0 + Line, data = Pheno)
K_g=Z_g%*% Geno%*%t(Z_g) ###Linear kernel

# Env design matrix without the first column
X_E<- model.matrix(~ 0 + Env, data = Pheno)[, -1]
K_e=X_E%*%t(X_E)/ncol(X_E)

X_g <-Z_g%*%Markers_Red
K_ge <- K_e* K_g
# Interaction matrix
X_ge<- model.matrix(~ 0 + X_g:X_E, data = Pheno)
dim(X_ge)

# Put the matrices in the expected format with the model
# to use with each one
X <- list(
  list(x =X_E, model = "FIXED"),
  list(x =X_g, model = "BRR"),
  list(x =X_ge, model = "BRR")
```

```
)
head(Pheno)
# Retrieve the two continuos response variables
y <- SKM::to_matrix(Pheno[, c("GY", "PHR")])

# Folds generation for random cross validation
# Folds generation for random line validation
folds <- cv_random_line(lines =as.character(Pheno$Line),folds_number=5,
testing_proportion =0.2)
#

# List for storing the individual predictions at each fold
Predictions <- list(GY = data.frame(), PHR = data.frame())

for (i in seq_along(folds)) {
  fold <- folds[[i]]

  # Set testing indices to NA in the response variables
  y_na <- y
  y_na[fold$testing, ] <- NA

  # *********** MODEL EVALUATION AND HYPERPARAMETERS TUNING WITH SKM
***********
  model <- SKM::bayesian_model(
    X,
    y_na,

    iterations_number = 10000,
    burn_in = 5000
  )

  # Predict over testing set
  predictions <- predict(model)

  for (trait in names(Predictions)) {
    # Save the predictions along with environment and line information
    Predictions[[trait]] <- rbind(
      Predictions[[trait]],
      data.frame(
        Fold = i,
        Line = Pheno$Line[fold$testing],
        Env = Pheno$Env[fold$testing],
        Observed = y[fold$testing, trait],
        Predicted = predictions[[trait]]$predicted
      )
    )
  }
}

# Errors metrics for prediction performance
```

```
GY_Summaries <- SKM::gs_summaries(Predictions$GY)

# Print summaries by line, environment and fold
GY_Summaries$line
GY_Summaries$env
GY_Summaries$fold

# Errors metrics for prediction performance
PHR_Summaries <- SKM::gs_summaries(Predictions$PHR)

# Print summaries by line, environment and fold
PHR_Summaries$line
PHR_Summaries$env
PHR_Summaries$fold
```

Example S5. Multivariate mixed variables

```
# Import SKM library
library(SKM)

# Load the dataset
load("Japonica_100.RData", verbose = TRUE)

# Oder Pheno by Env and Lines
Pheno <- Pheno[order(Pheno$Env, Pheno$Line), ]
Markers=Markers
geno_order <- sort(rownames(Markers))
Markers=Markers[geno_order ,]
Markers_Scale=scale(Markers[,-1])
dim(Markers_Scale)
Markers_Scale=Markers_Scale[ , colSums(is.na(Markers_Scale))==0]
dim(Markers_Scale)
Geno=Markers_Scale%*%t(Markers_Scale)/ncol(Markers_Scale)

# Order Geno by Line
Geno <- Geno[geno_order, geno_order]
Markers_SVD=svd(Markers_Scale)
dim(Markers_SVD$u)
Markers_Red=Markers_SVD$u%*%diag(Markers_SVD$d)
dim(Markers_Red)
# Design matrices
Z_g<- model.matrix(~ 0 + Line, data = Pheno)
K_g=Z_g%*% Geno%*%t(Z_g) ###Linear kernel

# Env design matrix without the first column
X_E<- model.matrix(~ 0 + Env, data = Pheno)[, -1]
K_e=X_E%*%t(X_E)/ncol(X_E)

X_g <-Z_g%*%Markers_Red
K_ge <- K_e* K_g
```

```
# Interaction matrix
X_ge<- model.matrix(~ 0 + X_g:X_E, data = Pheno)
dim(X_ge)
# Bind all matrices in a single one
X <- cbind(X_E, X_g, X_ge)


# Retrieve the two mixed response variables

Pheno$Cat=ifelse(Pheno$GY<quantile(Pheno$GY,probs=0.3),"Bottom",ifelse(Ph
eno$GY>quantile(Pheno$GY,probs=0.7),"Top","Middle"))
Pheno$Cat <- as.factor(Pheno$Cat)

y <- Pheno[, c("GY", "Cat")]

# Folds generation for stratified random cross validation
# it keeps the distribution of environment at each fold
folds <- SKM::cv_random_strata(
  data = Pheno$Env,
  folds_number = 5,
  testing_proportion = 0.25
)

# List for storing the individual predictions at each fold
Predictions <- list(DTHD = data.frame(), GY = data.frame())

for (i in seq_along(folds)) {
  fold <- folds[[i]]

  # Split training and testing data
  X_training <- X[fold$training, ]
  X_testing <- X[fold$testing, ]
  y_training <- y[fold$training, ]
  y_testing <- y[fold$testing, ]

  # *********** MODEL EVALUATION AND HYPERPARAMETERS TUNING WITH SKM
***********
  model <- SKM::deep_learning(
    X_training,
    y_training,

    # Tunable hyperparameters
    learning_rate = list(min = 0.001, max = 0.1),
    epochs_number = 50,
    batch_size = 32,
    layers = list(
      list(
        neurons_number = list(min = 20, max = 50),
        activation = "sigmoid",
        dropout = list(min = 0.2, max = 0.4)
```

```
    ),
    list(
      neurons_number = list(min = 20, max = 50),
      activation = "tanh",
      dropout = list(min = 0.2, max = 0.4)
    )
  ),

  # Tune configuration parameters
  tune_type = "Bayesian_optimization",
  tune_cv_type = "Random",
  tune_testing_proportion = 0.2,
  tune_folds_number = 2,
  tune_bayes_samples_number = 5,
  tune_bayes_iterations_number = 5,

  # Other algorithm's parameters
  optimizer = "adam",
  shuffle = TRUE,
  early_stop = TRUE,
  early_stop_patience = 10
)

# Predict over testing set
predictions <- predict(model, X_testing)

# Save the predictions along with environment and line information
Predictions$GY <- rbind(
  Predictions$GY,
  data.frame(
    Fold = i,
    Line = Pheno$Line[fold$testing],
    Env = Pheno$Env[fold$testing],
    Observed = y_testing$GY,
    Predicted = predictions$GY$predicted
  )
)

# Save the predictions along with environment and line information
Predictions$Cat <- rbind(
  Predictions$Cat,
  data.frame(
    Fold = i,
    Line = Pheno$Line[fold$testing],
    Env = Pheno$Env[fold$testing],
    Observed = y_testing$Cat,
    Predicted = predictions$Cat$predicted,
    predictions$Cat$probabilities
  )
)
```

```
}

# Errors metrics for prediction performance
GY_Summaries <- SKM::gs_summaries(Predictions$GY)

# Print summaries by line, environment and fold
GY_Summaries$line
GY_Summaries$env
GY_Summaries$fold

# Errors metrics for prediction performance
Cat_Summaries <- SKM::gs_summaries(Predictions$Cat)

# Print summaries by line, environment and fold
Cat_Summaries$line
Cat_Summaries$env
Cat_Summaries$fold
```

Example S6. Kernel model for an univariate continuous variable

```
# Import SKM library
library(SKM)

# Load the dataset
load("Japonica_100.RData", verbose = TRUE)

# Oder Pheno by Env and Lines
Pheno <- Pheno[order(Pheno$Env, Pheno$Line), ]
Markers=Markers
geno_order <- sort(rownames(Markers))
Markers=Markers[geno_order ,]
Markers_Scale=scale(Markers[,-1])
dim(Markers_Scale)
Markers_Scale=Markers_Scale[ , colSums(is.na(Markers_Scale))==0]
dim(Markers_Scale)
Geno=Markers_Scale%*%t(Markers_Scale)/ncol(Markers_Scale)

# Order Geno by Line
Geno <- Geno[geno_order, geno_order]
Markers_SVD=svd(Markers_Scale)
dim(Markers_SVD$u)
Markers_Red=Markers_SVD$u%*%diag(Markers_SVD$d)
dim(Markers_Red)
# Design matrices
Z_g<- model.matrix(~ 0 + Line, data = Pheno)
K_g=Z_g%*% Geno%*%t(Z_g) ###Linear kernel

# Env design matrix without the first column
X_E<- model.matrix(~ 0 + Env, data = Pheno)[, -1]
```

```
K_e=X_E%*%t(X_E)/ncol(X_E)

X_g <-Z_g%*%Markers_Red
K_ge <- K_e* K_g
# Interaction matrix
X_ge<- model.matrix(~ 0 + X_g:X_E, data = Pheno)
dim(X_ge)
# Bind all matrices in a single one
X <- cbind(X_E, X_g, X_ge)

X <- SKM::kernelize(
  X,
  kernel = "Sparse_Polynomial",
  rows_proportion = 0.75,
  gamma = 1 / ncol(X),
  coef0 = 0.5,
  degree = 4
)
# Retrieve the continuos response variable
y <- as.numeric(Pheno$GY)
str(y)

# Folds generation for k-fold cross validation
folds <- SKM::cv_kfold(nrow(Pheno), k = 5)

# Data frame for storing the individual predictions at each fold
Predictions <- data.frame()

for (fold_number in seq_along(folds)) {
  fold <- folds[[fold_number]]

  # Split training and testing data
  X_training <- X[fold$training, ]
  X_testing <- X[fold$testing, ]
  y_training <- y[fold$training]
  y_testing <- y[fold$testing]

  # *********** MODEL EVALUATION AND HYPERPARAMETERS TUNING WITH SKM
***********
  model <- SKM::random_forest(
    X_training,
    y_training,
    trees_number = c(100, 200),
    node_size = c(5, 2),

    tune_type = "Grid_search",
    tune_cv_type = "k_fold",
    tune_folds_number = 5,
    tune_loss_function = "mse"
  )
```

```
  # Predict over testing set
  predictions <- predict(model, X_testing)

  # Save the predictions along with environment and line information
  Predictions <- rbind(
    Predictions,
    data.frame(
      Fold = fold_number,
      Line = Pheno$Line[fold$testing],
      Env = Pheno$Env[fold$testing],
      Observed = y_testing,
      Predicted = predictions$predicted
    )
  )
}

# Errors metrics for prediction performance
Summaries <- SKM::gs_summaries(Predictions)

# Print summaries by line, environment and fold
Summaries$line
Summaries$env
Summaries$fold
```
